# Lateral tarsal strip procedure for involutional ectropion: A retrospective analysis of 85 cases and a comprehensive literature review

**DOI:** 10.1016/j.aopr.2021.100004

**Published:** 2021-09-09

**Authors:** Xiaoyi Hou, Yongwei Guo, Senmao Li, Ming Lin, Renbing Jia, Alexander Rokohl, Ludwig M. Heindl

**Affiliations:** a Department of Ophthalmology, West China Hospital of Sichuan University, Chengdu, China; b Department of Ophthalmology, Faculty of Medicine and University Hospital Cologne, University of Cologne: Universitat zu Koln, Germany; c Eye Center, Second Affiliated Hospital, School of Medicine, Zhejiang University, Hangzhou, China; d Department of Ophthalmology, Ninth People’s Hospital, Shanghai Jiao Tong University, Shanghai, China; e Center for Integrated Oncology (CIO) Aachen-Bonn-Cologne-Duesseldorf, Cologne, Germany

**Keywords:** Involutional ectropion, Aging eyelid, Lateral tarsal strip, Lower eyelid tension, Post-operative surgical effects

## Abstract

**Purpose:**

To evaluate the postoperative effect of the lateral tarsal strip (LTS) procedure in treating lower eyelid involutional ectropion.

**Methods:**

A retrospective study was performed on 85 eyelids in 67 patients with involutional ectropion who underwent LTS procedure. Pre- and postoperative lower eyelid laxity and ocular symptoms as well as perioperative complications were evaluated. Snap back test was performed to evaluate the elasticity of lateral canthal tendon loosening and, a lower lid distraction test was performed to evaluate the degree of severity before surgery. Ocular surface diseases were evaluated by the Ocular Surface Disease Index (OSDI), and symptoms including conjunctivitis, corneal ulcer, dry eye syndrome, and ocular pain were recorded. All patients were evaluated within one week and during the follow-up period of 4.2 ​± ​8.3 months. Furthermore, we reviewed the studies that also investigated the surgical effect of the LTS procedure in the literature from 1979 to 2019.

**Results:**

The success rate was 95%. Only four eyelids required a second surgical intervention. Seventy-three (86%) eyelids had an excellent position after surgery, 9 (11%) only little improvement, and three had no improvement. No significant difference was found in the postoperative effects between different degrees of ectropion (p ​> ​0.05). No statistical correlation was found between surgical improvements and the ectropion severity (P ​> ​0.05). Fifty-two out of 85 eyes had no discomfort after the surgery. Mild complications included epiphora in 13 eyes (three cases caused by lacrimal punctum eversion), ocular pain in 12 eyes, wound hemorrhage in 12 eyes, and edema in 9 eyelids immediately after surgery, in which 91.2% (n ​= ​21) disappeared within one week and did not need any further treatment.

**Conclusions:**

The lateral tarsal strip procedure can provide an aesthetically pleasing result for correcting the mild to moderate lower eyelid ectropion while maintaining decent eyelid function.

## Highlights

• A safe, effective, and economical option for involutional ectropion.
• Decent aesthetical and functional improvement after lateral tarsal strip procedure.
• The first review for lateral tarsal strip in correcting involutional ectropion.
• Comprehensive treatment options of ectropion for oculoplastic surgeons.

## 1 Introduction

Involutional ectropion is the main form of lower eyelid ectropion, which is due to the imbalanced forces with increased age^1^ and gradual lower eyelid laxity under the influence of loosening eyelid skin, weakened orbicularis muscles, decreased medial and lateral canthus tension, as well as altered eyelid gravity.^2^^,^^3^ Generally, lateral canthal tendon (LCT) laxity or extension is the main reason of most paralytic or involutional upper and lower eyelid laxity, lateral canthus malposition, and iatrogenic retraction associated with postoperative recurrent entropion or ectropion^4^^,^.^5^ Malposition of the lower eyelid with untreated ectropion can cause lacrimal punctum eversion, conjunctiva, and cornea exposure, which may eventually lead to ocular surface damage resulting in photophobia, eye-watering, infections, vision loss, and ocular pain.^6^, ^7^, ^8^

Lateral tarsal strip (LTS) was initially introduced by Anderson et al. in 1979^1^^,^^4^, to correct lower lid laxity including paralytic or senile malposition. The upward and inward tension of the lower eyelid increased by shortening the lateral tarsus and reconstructing the attachment point of the LCT.^9^^,^^10^ It can effectively antagonize both vertical retraction and horizontal eversion, and restore the normal eyelid anatomy and function especially for the abnormal position of the lower lid and the lateral canthus.^11^ Due to the wide range of indications, LTS is currently the most commonly used method for correcting the lower eyelid laxity including involutional ectropion.^12^ However, its efficiency in correcting lower eyelid ectropion when performed independently is still controversial, especially in patients with epiphora or partial punctum stenosis.^13^ Hence, in our study, we aimed to evaluate the clinical effect of the LTS procedure for correcting involutional lower eyelid ectropion with or without epiphora.

## 2 Materials and methods

### 2.1 General information

We included patients with involutional lower eyelid ectropion who felt ocular discomfort when eyes opened and underwent the standard LTS surgery^14^ from January 2013 to January 2018 ​at the Department of Ophthalmology, University of Cologne. All cases with a spastic, cicatricial, or traumatic ethology, as well as ectropion secondary to facial nerve palsy, were excluded. Afterward, basic information of all cases including gender, eye side, age, follow-up period, preoperative symptoms, and ophthalmic examination was recorded. All eyes underwent a comprehensive ophthalmological slit-lamp examination, especially for lacrimal punctal eversion, palpebral conjunctiva congestion, and corneal spotty or flaky staining (Table 1).

**Table 1:** General information of patients.

Categories		Count (Percentage)
Age range (year)		58∼98 (80.9 ​± ​8.2)
Sex (Patients' no.)	Male	46 (68.7%)
	Female	21 (31.3%)
	Total	67
Follow-up (Month)		0∼45 (4.2 ​± ​8.3)
Eyelids (No.)	Right lower eyelid	40 (47.1%)
	Left lower eyelid	45 (52.9%)
	Total	85
Degree of ectropion (Eyelids)	Mild	24 (28.2%)
	Moderate	28 (32.9%)
	Severe	31 (36.5%)
	Extreme	2 (2.4%)
	Total	85
Preoperative symptoms (affected eyes)	Corneal ulcer	2 (2.2%)
	Ocular pain	2 (2.2%)
	Dry eye syndrome	4 (4.5%)
	Conjunctivitis	7 (7.8%)
	Epiphora	21 (23.6%)
	Total	36 (40.3%)

The study protocol was approved by the ethics review board of the University of Cologne. All of the procedures were performed in accordance with the Declaration of Helsinki and relevant policies in Germany. Because of the retrospective nature of the study, patient consent for inclusion was waived.

### 2.2 Evaluation index

#### 2.2.1 Pre-operative assessment

Patients with a medical history that may lead to a reactional hemorrhage were excluded, e.g., uncontrolled hypertension, bleeding diathesis, thyrotoxicosis, arteriosclerosis, and concurrent aspirin or anticoagulants predispose. Ectropion that resulted from previous eye disease or trauma was also excluded.

Snap back test (SBT) was performed to evaluate the elasticity of LCT loosening.^9^ Normally, the lower eyelid should return to the globe immediately in SBT, which was graded as positive if any delay of the lower eye lid return was clinically noted. Additionally, lid distraction test (DT) was conducted to evaluate the degree of severity^9^^,^^10^ before surgery by pulling lower eyelid away from the globe. The normal distraction distance is 7–8 ​mm or less, and the result was defined as positive if any increase in the distraction distance was recognized.

Only eyelid margin eversion (medial or lateral) without any ocular symptoms was defined as mild ectropion; lower eyelid eversion with the exposure of underlying sclera with mild symptoms such as dry eye syndrome and redness was defined as moderate ectropion; lower eyelid eversion with the exposure of lower conjunctiva or entire lower eyelids isolated from the eyeball with mild symptoms such as epiphora and conjunctivitis was defined as severe ectropion; severe lower eyelid ectropion together with exposure keratitis or even corneal ulcer was defined as extreme ectropion (Table 2).

**Table 2:** Grading for lower eyelid ectropion severity.

Categories	Description
Mild	Eyelid margin eversion (medial or lateral) without any ocular symptoms
Moderate	Lower eyelids eversion with the exposure of underlying sclera with mild symptoms such as dry eye syndrome and redness
Severe	Lower eyelids eversion with the exposure of lower conjunctiva or entire lower eyelids isolated from the eyeball with mild symptoms such as epiphora and conjunctivitis
Extreme	Severe lower eyelids ectropion together with exposure keratitis or even corneal ulcer

#### 2.2.2 Post-operative evaluation

Post-operative effects were categorized using a semiquantitative grading score: better, the same, and worse.^15^^,^^16^ The severity level of the lower eyelid laxity was graded according to the manifestation of lower eyelids. Only eyelid margin eversion (medial or lateral) was graded as mild ectropion while exposure of underlying sclera or exposure of inferior palpebral conjunctiva was defined as moderate. The full isolation of the eyelid from the eyeball with mild symptoms such as conjunctivitis was graded as severe; an extreme ectropion was defined as severe lower eyelid eversion accompanied by exposure keratitis or even corneal ulcer.^16^, ^17^, ^18^ For the functional improvement, the improvement of ocular surface diseases was evaluated by the Ocular Surface Disease Index (OSDI), and symptoms including conjunctivitis, corneal ulcer, dry eye syndrome, and ocular pain were recorded.

#### 2.2.3 Theory/surgical technique

**Fig. 1:**
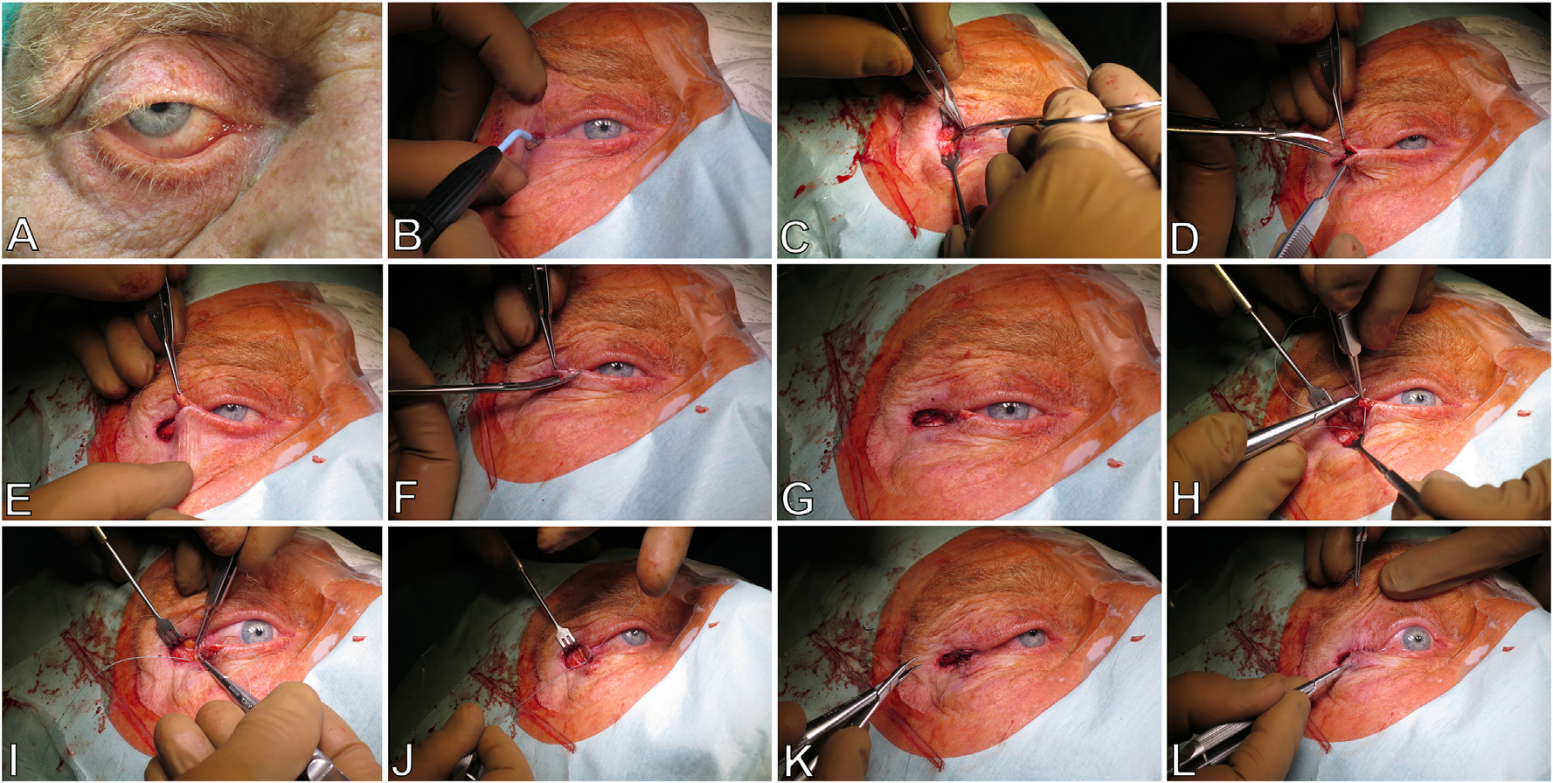
Operation procedure of lateral tarsal strip. A. Pre-operative figure of an 85-year-old man. B. Lateral canthotomy.C. Separation of the anterior and posterior lamella of through the gray line. D-E. Mimic post-operative effects and excision of excess tarsal strip and eyelid skin. F. Finish reparation of the tarsal strip. G-H. Puncture through the tarsal strip and fix it onto the periosteum. I-J. Tighten the sutures and fix the ligation. K. Reconstruction of the lateral canthal angle. L. Suture the skin incision.

All surgeries were performed by one surgeon (LMH) in a standardized fashion. For local anesthesia, 3–4 ​ml of a mixture of 2% lidocaine and 1:100.000 epinephrine was injected using a 27-gauge needle superficially into the lateral eyelid, the lateral canthal angle, and deeply into the lateral orbital rim region (Fig. 1A). To promote hemostasis, a horizontal skin incision was made with monopolar from the lateral canthus to the orbital rim (Fig. 1B). With the help of a pull hook, the incision was deepened enough to expose the orbital rim (Fig. 1C). Then, the lateral canthotomy was made to release the lower lid using surgical scissors, i.e., transecting the inferior branch of the lateral canthus ligament attached to the lateral orbital rim. The anterior and posterior lamella of the lower eyelid were separated through the grayline (Fig. 1D). The skin and orbicularis muscle were excised and the palpebral conjunctiva of the caudal margin was scrapped to prepare the tarsal strip. Under gentle traction, the strip was pulled over the lateral canthal angle to simulate the surgical effect (Fig. 1E). Part of the excess skin was excised triangularly to help the lower lid cling to the eyeball (Fig. 1F–G). The suture between the lateral periosteum and the tarsal strip was performed using a double-ended non-absorbable suture (5.0 Ethibond) with a half-circle needle (Fig. 1H). The needle was inserted into the periosteum from an anterior to a posterior direction vertical to the lateral bone (Fig. 1I). Then a full-thickness puncture was made by the same needle passing into the anterior surface of the tarsal strip in the lower edge, which was punctured back to the upper edge of the tarsal strip from the posterior surface and was directly inserted into the lateral periosteum from a posterior to the anterior direction parallel to the prior suture placement (Fig. 1J). The two groups of non-absorbable sutures were tightened, and the lower eyelid margin was observed to be attached to the corneal limbus (Fig. 1K–L). Then, the ligation was fixed, the skin margin of the upper eyelid was trimmed, and the Vicryl 5.0 suture was passed through the gray lines of upper and lower lid margins to reconstruct the lateral canthal angle. Finally, the skin incision was trimmed and sutured. Pressure bandaging lasted for one day.

### 2.3 Statistical analyses

Commercial software (SPSS version 23.0 for IOS; IBM Corp., Armonk, N.Y., USA) was used for all statistical analyses. A chi-squared test was used to analyze the statistical significance of abnormal-distributed valuables. Spearman correlation was used to test the correlation between the surgical improvements and the severity of ectropion. *P-value* < 0.05 has statistical significance.

## 3 Results

**Fig. 2:**
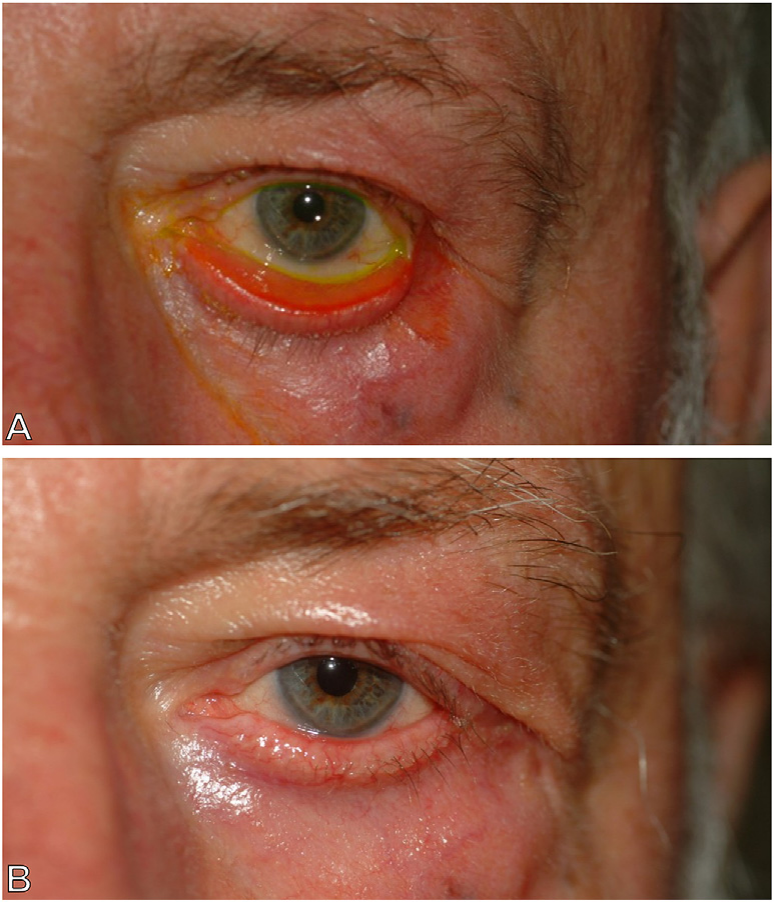
The pre- and post-operative picture of an 87-year-old man. A. An 87-year-old man presented with involutional ectropion of the left lower eyelid in conjunction with lower eyelid laxity. B. After standard lateral tarsal strip procedure, function and appearance were well improved.

A total of 67 patients (46 males and 21 females) with involutional ectropion (Fig. 2A) underwent standard LTS surgery (Fig. 2B) in our study from January 2013 to January 2018. A total of 85 eyelids with different degrees of ectropion underwent standard LTS surgery, including 24 mild ectropion (28%), 28 moderate ectropion (33%), 31 severe ectropion (37%), and 2 extreme ectropion (2%). A total of 36 affected eyes had pre-operative symptoms including 21 (23.6%) with epiphora, 2 (2.2%) with corneal ulcer, 2 (2.2%) with ocular pain, 4 (4.5%) with dry eye syndrome, and 7 (7.8%) with conjunctivitis. The average follow-up period is 4.2 ​± ​8.3 (0 ∼ 45) months (Table 1).

The definition of grading the severity of lower eyelid ectropion was shown in Table 2. The post-operative improvements for different severity of lower eyelid ectropion were shown in Table 3, respectively. Seventy-three (86%) eyelids had an excellent position after surgery, nine (11%) eyelids had only little improvement, and three eyelids had no improvement. No statistical significance of post-operative effects was found between different degrees of ectropion (p ​> ​0.05). No statistical correlation was found between surgical improvements and the ectropion severity (p ​> ​0.05).

**Table 3:** Post-operative anatomical improvements for different severity of lower eyelid ectropion.

Patients'number (Percentage)	Post-operative improvements				
	Grade	Better	Worse	Same	Total
Lower lids ectropion	Mild	18 (75.0%)	2 (8.3%)	4 (16.7%)	24
	Moderate	26 (92.9%)	0	2 (7.1%)	28
	Severe	27 (87.1%)	1 (3.2%)	3 (9.7%)	31
	Extreme	2 (100%)	0	0	2
	Total	73 (85.9%)	3 (3.5%)	9 (10.6%)	85

The post-operative functional improvements for pre-existing symptoms were shown in Table 4. *A total of* *21* *patients had pre-existing epiphora, which is caused by lacrimal punctum eversion.* Nineteen out of 21 patients with pre-existing epiphora had significant improvement. One patient did not feel any improvement, and another patient felt even worse right after the surgery but gradually improved during the follow-up period. Ten (83%) patients with preoperative symptoms were also significantly relieved and remained in the later follow-up period, and only 2 patients felt no improvement or even worse than before the surgery.

**Table 4:** Post-operative functional improvements for pre-existing symptoms.

Functional Improvements	Grade	Patients'number (Percentage)
Epiphora	Better	19 (90.5%)
	Worse	1 (4.8%)
	The same	1 (4.8%)
	Total	21
Other symptoms	Better	10 (83.3%)
	Worse	1 (8.3%)
	The same	1 (8.3%)
	Total	12

The postoperative complications were shown in Table 5. Fifty-two (61.2%) out of 85 eyes had no discomfort after surgery. Thirteen (15.3%) eyes still had epiphora after surgery with lacrimal punctum eversion in three patients. Twelve (14.1%) eyes were with ocular pain, 12 (14.1%) with wound hemorrhage, and 9 (10.6%) with eyelids edema immediately after the surgery, but disappeared in all cases within one week and did not need any further treatment. There were five patients with moderate complications, three (3.5%) of them had lacrimal punctum eversion accompanied by epiphora, one (1.1%) had an overcorrection, and one (1.1%) patient had mild wound infection. Four (4.7%) eyelids from three patients developed recurrence and needed a second surgery to correct the residual ectropion after surgery. One of them had an ectropion formed by a scarring wound and underwent a local loosening. The other two patients had an insufficient correction of ectropion and needed a re-adaptation of the placement between the tarsal strip and the peri-orbit. After a second surgery, all of them had an ideal eyelid position, and the surgical effect remained stable in the follow-up period.

**Table 5:** Post-operative complications.

Grade	Category	Eyelids (Percentage)
No complications		52 (61.2%)
Mild	Epiphora	13 (15.3%)
	Hemorrhage	12 (14.1%)
	Edema	9 (10.6%)
	Pain	12 (14.1%)
Moderate	Lacrimal punctum eversion	3 (3.5%)
	Eyelid overcorrected	1 (1.1%)
	Wound infection	1 (1.1%)
Severe	Moderate to severe ectropion still need additional surgery	4 (4.7%)

## 4 Discussion

In this study, we analyzed the postoperative outcome of using standard LTS to correct the involutional ectropion, based on preoperative and postoperative photographs and the records of symptoms. Furthermore, we used a semiquantitative grading score (better, the same, and worse) to categorize the post-operative improvements. The results demonstrated that mild to moderate lower eyelid involutional ectropion may be aesthetically corrected, and decent eyelid function in patients with epiphora may also be maintained after LTS procedure. In addition, to the best of our knowledge, this is the first literature review for the surgical effect of LTS procedure in correcting involutional ectropion.

After correcting by standard LTS, 97% of the affected eyelids got anatomical improvement, and only three eyelids had no improvement on the eyelid position. Additionally, the majority of patients with pre-existing epiphora and other symptoms had significant improvements after surgery and observed stability within the follow-up period, which indicated that the LTS could be an effective and reliable way of correcting involutional ectropion with pre-existing epiphora when performed in isolation.

No significant difference was found in postoperative effects between mild and moderate degrees of ectropion, and no statistical correlation was found between surgical improvements and the ectropion severity, which was consistent with previous study^14^ that suggested LTS can effectively correcting ectropion from mild to moderate level. Additionally, according to our results, an improvement of severe involutional ectropion by correcting with isolated LTS was possible. However, only two severe cases were included in our current study. Hence, this finding does not exclude individual cases that need to be corrected by additional or combined surgery methods.^19^ The original LTS procedure^4^ was first reported in 1979 with a wide range of indications including involutional and paralytic eyelid malposition and was enhanced by Jordan, D. R. et al.^20^ in 1989 for correcting paralytic ectropion, suggesting a modification of original LTS and lower eyelid retraction could also be treated (Supplementary Table 1). Although different surgeries^21^, ^22^, ^23^, ^24^, ^25^, ^26^, ^27^, ^28^ were reported to correct lower eyelid ectropion successfully, LTS is still the optimum procedure to correct the lateral canthus ligament relaxation.^21^, ^22^, ^23^, ^24^, ^25^, ^26^, ^27^, ^28^ Variant modifications over the primary LTS procedure were investigated up to date.^29^^,^^30^ A modified LTS was introduced by Lopez-Garcia, J. S. et al.^29^ to improve the outcomes, reduce the complications and recurrence rates.^29^ However, this modification was recommended for involution entropion with a severe loss of the lower lid retractor. Jones procedure^31^, ^32^, ^33^ was also recommended for vertical lower lid laxity, which plicates the lower lid retractors to tighten the capsule palpebral fascia and as a result, the vertical traction power of the lower lid ligaments was reinforced. It is an effective and economical option but more recommended for patients with senile lower lid entropion. In the modified LTS from Meduri, A. et al.,^30^ a double suture was placed in the medial part of the tarsal strip and the conjunctival cuts were performed to reduce the high recurrence rates in severe ectropion. Sommer, F. et al.^34^ investigated the surgical results of using an absorbable or nonabsorbable periosteal fixation for the tarsal strip, nevertheless, no significant surgical outcome was found between these sutures in the lower lid ectropion.

The lower eyelid is important in protecting the globe and lubricating the eye while aiding in the drainage of tears. Disruption of the lower eyelid in the form of ectropion can result in eversion of the lacrimal punctum with exposure of the conjunctiva and cornea and can lead to epiphora, photophobia, conjunctival infection, decreased vision, and pain of the ocular surface. In our study, the majority of the patients showed no complication or mild complications including epiphora, wound bleeding, eyelid edema, or wound pain, which could disappear within the next three days. The moderate and less accompanying complications were lacrimal punctum eversion uncorrected and over- or under-correction, which could be corrected again by a second surgery, which was also consistent with the previous study^13^ indicating that LTS alone could be sufficient for most case except. Furthermore, although three patients in our study were with residual lacrimal punctum eversion after the LTS procedure, no additional surgery were performed as they showed enough functional improvement, e.g., epiphora and dry eye syndrome (Table 4). In the study of Ehrhardt, A. et al.,^13^ the LTS procedure with three snip punctoplasty was firstly introduced to compare with LTS alone, to evaluate the effectiveness in reducing epiphora. However, no greater reduction in eyewatering was found, and hence, LTS alone was suspected to be sufficient in most cases of involutional ectropion with eyewatering.^13^ In addition, LTS with a simultaneous tarsoconjunctival dia-mond excision (medial spindle) was suggested to apply in refractory ectropion such as cicatricial ectropion secondary to eyelid tumor excision.^8^

Korteweg et al.^35^ developed a highly reliable grading system, the Ectropion Severity Score (ESS), to quantify the severity of patients with paralytic ectropion seen on photographs. The inter-rater reliability of ESS was proved to be sensitive for evaluating the existence of ectropion. However, in their research, a formal validity test was not performed. In the prospective study of Moe et al.,^17^ an Ectropion Grading Scale (EGS) was developed to evaluate the type and severity of ectropion caused by paralysis, which was not corrected by LTS. Although these studies^17^^,^^18^^,^^35^ have investigated the grading of lower eyelid ectropion, there is currently no internationally unified scoring standard for the retrospective analysis on involution ectropion. Hence, in our study, we use a grading semi-quantitative score (better, the same, worse) to record the anatomical and functional improvement after the standard LTS, which was proved to be simple and sensitive to record the surgical outcome of involutional ectropion.

The possible limitation of this study might be the grading of the disease severity only based on the pre- and post-operative photographs and the missing part of a control group to compare the surgical outcome with our technique.

In summary, our results demonstrated that the LTS procedure can provide an aesthetically pleasing result for correcting the mild to moderate lower eyelid ectropion. LTS can effectively maintain decent eyelid function in patients with epiphora or partial punctum stenosis. Hence, the LTS procedure could be a further improvement as an excellent, time-efficient method that is the definitive choice of treatment for numerous eyelid disorders that need horizontal eyelid tightening. Furthermore, our comprehensive review of LTS procedure will provide oculoplastic surgeons a comparative understanding of the treatment option of ectropion.

## Study Approval

All subjects gave their informed consent for inclusion before they participated in the study. The study was conducted in accordance with the Declaration of Helsinki, and the protocol was approved by the Ethics Committee of University of Cologne (approval number:17–199).

## Author Contributions

XY H, YW G, and LM. H conceived and designed the study; XY H and SM L performed the study; All authors analyzed the data; A. R and LM. H contributed materials and evaluation; XY H wrote the paper, and all authors revised the paper. All authors have read and approved the manuscript.

## Acknowledgments

This work thanks to Ms. Fabiana Salati for her contribution of collecting the patients' data.

## Funding

This work was supported by the 10.13039/501100012226Fundamental Research Funds for the Central Universities (grant No. 2021FZZX005-15); the Koeln Fortune Program/Faculty of Medicine, 10.13039/501100008001University of Cologne, Germany (grant No. 2680148101); 10.13039/501100001809National Natural Science Foundation of China (grant No. 81970834); 10.13039/501100003399Science and Technology Commission of Shanghai Municipality (grant No. 19441900800) and GEROK program of the 10.13039/501100008001University of Cologne.

## Conflict of Interest

The authors declare that they have no known competing financial interests or personal relationships that could have appeared to influence the work reported in this paper.

## Abbreviations

LTS: lateral tarsal strip
ESS: ectropion severity score
EGS: ectropion srading scale
MS: medial spindle
MLTS: modified tarsal strip
CLTS: conventional LTS
TB: tarsal belt
QS: quickert sutures
DCR: endonasal dacryocystorhinostomy
HPMG: hard palate mucosa grafts
PE: paralytic ectropion
IEn: involutional entropion
IEc: involutional ectropion
PEc: punctal ectropion
IPEc: involutional punctal ectropion
HEL: horizontal eyelid laxity
IMEc: involutional medial ectropion
PE: punctal eversion
LCT: lateral canthal tendon
DT: distraction test
SBT: snap back test
OSDI: ocular surface disease index

## References

[1] Anderson R.L. (1981). Tarsal strip procedure for correction of eyelid laxity and canthal malposition in the anophthalmic socket. Ophthalmology.

[2] Jordan D.R., Anderson R.L. (1989). The lateral tarsal strip revisited. The enhanced tarsal strip. Arch Ophthalmol.

[3] Tucker S.M., Santos P.M. (1999). Survey: management of paralytic lagophthalmos and paralytic ectropion. Otolaryngol Head Neck Surg.

[4] Anderson R.L., Gordy D.D. (1979). The tarsal strip procedure. Arch Ophthalmol.

[5] Chan J.B., Looi A.L. (2014). The Looi suture technique for anchoring the lateral tarsal strip to the lateral orbital wall. Ann Acad Med Singapore.

[6] Frueh B.R., Su C.S. (2002). Medial tarsal suspension: a method of elevating the medial lower eyelid. Ophthalmic Plast Reconstr Surg.

[7] Wright M., Bell D., Scott C. (1999). Everting suture correction of lower lid involutional entropion. Br J Ophthalmol.

[8] Lee H., Park M., Chang M. (2015). Clinical characteristics and effectiveness of the lateral tarsal strip and medial spindle procedure. Ann Plast Surg.

[9] Weinstein G.S., Anderson R.L., Tse D.T. (1985). The use of a periosteal strip for eyelid reconstruction. Arch Ophthalmol.

[10] Game J., Morlet N. (2007). Lateral canthal fixation using an oblique vertically orientated asymmetric periosteal transposition flap. Clin Exp Ophthalmol.

[11] Eshraghi B., Jamshidian-Tehrani M., Fadakar K. (2018). Vector analysis of changes in corneal astigmatism following lateral tarsal strip procedure in patients with involutional ectropion or entropion. Int Ophthalmol.

[12] Vahdani K., Ford R., Garrott H. (2018). Lateral tarsal strip versus Bick's procedure in correction of eyelid malposition. Eye.

[13] Ehrhardt A., Guechi O., Zaidi M. (2018). Lateral tarsal strip versus lateral tarsal strip with three-snip punctoplasty for managing epiphora in involutional ectropion. J Fr Ophtalmol.

[14] Kam K.Y., Cole C.J., Bunce C. (2012). The lateral tarsal strip in ectropion surgery: is it effective when performed in isolation?. Eye.

[15] Olver J.M., Barnes J.A. (2000). Effective small-incision surgery for involutional lower eyelid entropion. Ophthalmology.

[16] Olver J.M., Sathia P.J., Wright M. (2001). Lower eyelid medial canthal tendon laxity grading: an interobserver study of normal subjects. Ophthalmology.

[17] Moe K.S., Linder T. (2000). The lateral transorbital canthopexy for correction and prevention of ectropion: report of a procedure, grading system, and outcome study. Arch Facial Plast Surg.

[18] Pascali M., Corsi A., Brinci L. (2014). The tarsal belt procedure for the correction of ectropion: description and outcome in 42 cases. Br J Ophthalmol.

[19] Fong K.C., Mavrikakis I., Sagili S. (2006). Correction of involutional lower eyelid medial ectropion with transconjunctival approach retractor plication and lateral tarsal strip. Acta Ophthalmol Scand.

[20] Mahe E., Harfaoui-Chanaoui T., Banal A. (1989). Different technical approaches for blepharoplasty in eyelid rejuvenation surgery. Arch Oto-Rhino-Laryngol.

[21] Awotesu S., Dubois V., El-Hindy N. (2010). Jessner's lymphocytic infiltrate: a rare cause of lid ectropion. BMJ Case Rep.

[22] Patel B.C., Patipa M., Anderson R.L. (1997). Management of postblepharoplasty lower eyelid retraction with hard palate grafts and lateral tarsal strip. Plast Reconstr Surg.

[23] Rougraff P.M., Tse D.T., Johnson T.E. (2001). Involutional entropion repair with fornix sutures and lateral tarsal strip procedure. Ophthalmic Plast Reconstr Surg.

[24] Hsuan J., Selva D. (2004). The use of a polyglactin suture in the lateral tarsal strip procedure. Am J Ophthalmol.

[25] Pascali M., Avantaggiato A., Carinci F. (2017). Tarsal strip versus tarsal belt in ectropion correction: a statistical evaluation. J Craniofac Surg.

[26] Dunbar K.E., Cox C., Heher K.L. (2017). Lateral tarsal strip plus skin-muscle flap excision in the treatment of lower eyelid involutional entropion. Orbit.

[27] Ghafouri R.H., Allard F.D., Migliori M.E. (2014). Lower eyelid involutional ectropion repair with lateral tarsal strip and internal retractor reattachment with full-thickness eyelid sutures. Ophthalmic Plast Reconstr Surg.

[28] Ho S.F., Pherwani A., Elsherbiny S.M. (2005). Lateral tarsal strip and quickert sutures for lower eyelid entropion. Ophthalmic Plast Reconstr Surg.

[29] Lopez-Garcia J.S., Garcia-Lozano I., Gimenez-Vallejo C. (2017). Modified lateral tarsal strip for involutional entropion and ectropion surgery. Graefes Arch Clin Exp Ophthalmol.

[30] Meduri A., Inferrera L., Oliverio G.W. (2018). The use of a double suture and conjunctival cuts in the lateral tarsal strip: a new approach to involutional ectropion. J Craniofac Surg.

[31] Jones L.T., Reeh M.J., Wobig J.L. (1972). Senile entropion. A new concept for correction. Am J Ophthalmol.

[32] Lessa S., Carreirao S. (1980). A simple method for the correction of senile entropion. Ann Plast Surg.

[33] Jones I.S., Cooper W.C. (1973). Lateral canthal reconstruction. Trans Am Ophthalmol Soc.

[34] Sommer F. (2017). [Surgery of lower eyelid ectropion with the tarsal strip procedure, using absorbable or non-absorbable sutures for periosteal fixation]. Klin Monbl Augenheilkd.

[35] Korteweg S.F.S., Stenekes M.W., van Zyl F.E. (2014). Paralytic ectropion treatment with lateral periosteal flap canthoplasty and introduction of the ectropion severity score. Plast Reconstr Surg Glob Open.

