# Supplementary material for: Lateral tarsal strip procedure for involutional ectropion: A retrospective analysis of 85 cases and a comprehensive literature review
Source: Adv Ophthalmol Pract Res. 2021 Sep 9;1(1):100004. doi: 10.1016/j.aopr.2021.100004 (PMC10577831; doi:10.1016/j.aopr.2021.100004)
Supplement: Multimedia component 1 [file mmc1.docx]

Supplementary Table 1: Literature review of Lateral Tarsal Strip Since 1979

| **First Author** | **Year** | **Eyes** | **Procedures** | **Age (Years)** | **Study Design** | **Disease** | **Reoperation rate for LTS** | **Follow-up (months)** |
| --- | --- | --- | --- | --- | --- | --- | --- | --- |
| Eshraghi, B. et.al^33^ | 2019 | 19 | LTS | 82.6 ± 5.2 | prospective nonrandomized interventional case  series | IEc or IEn | - | 3 months |
| Vahdani, K. et.al^12^ | 2018 | 641 | LTS v.s. Bick's | 76.1 ± 11 | Retrospective | Involutional or recurrent ectropion and entropion, Lax eyelid syndrome, Floppy eyelid syndrome | 9% | 13.07 ± 7, (0.5∼58) |
| Meduri, A. et.al^30^ | 2018 | 16 | MLTS | 69.5 ± 6.2 | prospective randomized study | symptomatic severe IEc | 0 | 18 |
| Ehrhardt, A. et.al^13^ | 2018 | 40 | LTS v.s. LTS with three-snip punctoplasty | 79±11 | prospective, comparative, single center study | IEc with partial punctal stenosis | 0 | 3 |
| Sommer, F. et.al^31^ | 2017 | 148 | LTS+different suture | - | prospective | IEc | - | 6 |
| Pascali, M. et.al^25^ | 2017 | 64 | LTS v.s. TB | 56(38∼80) | retrospective | IEc | - | 24 |
| Lopez-Garcia, J. S. et.al^29^ | 2017 | 184 | MLTS v.s. CLTS | - | prospective study | IEn; IEc | CLTS (17.4 %), MLTS (4%) | 65 |
| Jue, M. S. et.al^34^ | 2017 | 44 | LTS | 71.4±7.9(53∼87) | Retrospective study | PEc with Leprosy | 11% | 12 |
| Dunbar, K. E. et.al^26^ | 2017 | 93 | LTS + skin-muscle flap excision v.s. QS | - | Retrospective Studies | IEn | 1% | 16.7 |
| Lee, H. et.al^8^ | 2015 | 111 | LTS v.s. MS | 61.5 (24∼82) | Retrospective Studies | IEc; IEn; IPEc | Simultaneous procedure LTS (86.5%) MS (92.7%) | 6 |
| Korteweg, S. F. et.al^35^ | 2014 | 30 | LTS + Lateral periosteal flap | 65 (36∼85) | A cross-sectional outcome study | PEc | 13%  - | 24 |
| Ghafouri, R. H. et.al^27^ | 2014 | 41 | LTS + internal retractor reattachment with full-thickness eyelid sutures | 82.2 ± 5.9(69 ∼92) | Retrospective review | IEc | - | 5.9 |
| Lee, H. et.al^36^ | 2013 | 241 | DCR ± LTS ± MS | 63.8±10.0 | Retrospective, interventional | IPEc; HEL; nasolacrimal | 12.6 % in DCR, 7.0 % in D + T, 13.0 % in D + M,  14.3 % in D + M + T | 5.6±3.3 |
| Kam, K. Y. et.al^18^ | 2012 | 67 | LTS v.s. LTS +MS | 76 (69∼82) | Retrospective Studies | IEc±PE | 13% | 6 (2∼12) |
| Fong, K. C. et.al^19^ | 2006 | 24 | LTS + Transconjunctival approach retractor plication | 79.7 | Prospective Studies | IMEc | 0 | 12 |
| Ho, S. F. et.al^28^ | 2005 | 41 | LTS + QS | 76.67 | Noncomparative interventional case series | IEn | 12.2% | 24 |
| Hsuan, J. et.al^24^ | 2004 | 105 | LTS + Polyglactin suture | 78(46∼92) | Prospective, interventional, consecutive case  series. | IEc; IEn | 13,3% | 9.1(3 to 32) |
| Rougraff, P. M. et.al^23^ | 2001 | 152 | LTS + Fornix sutures | 75 | Retrospective Studies | IEn | 1.6% | 36 |
| Patel, B. C. et.al^22^ | 1997 | 29 | LTS v.s. HPMG | - | - | lamella scarring; HEL | - | 14(6 to 30) |
| Jordan, D. R. et.al^20^ | 1989 | 44 | LTS | 71.4±7.9 (53∼87) | Retrospective study | PEc | 11.4% | 12 |
| Becker, F. F. et.al^37^ | 1982 | 6 | LTS | - | - | PEc | 66.7% | - |
| Anderson, R. L. et.al^4^ | 1979 | 1 | LTS | 71 | Case study | PEc | 0 | 12 |

Abbreviations: LTS, lateral tarsal strip; MS, medial spindle; MLTS, modified tarsal strip; CLTS, conventional LTS; TB, tarsal belt; QS, quickert sutures; DCR, endonasal dacryocystorhinostomy; HPMG, hard palate mucosa grafts; PE, paralytic ectropion; IEn, involutional entropion; IEc, involutional ectropion; PEc, punctal ectropion; IPEc involutional punctal ectropion; HEL; horizontal eyelid laxity; IMEc, involutional medial ectropion; PE, punctal eversion.
